# Associations across 22 dental and craniovertebral anomalies or variations, sagittal skeletal relationships, and vertical growth patterns: a comprehensive epidemiological study of 43 dentoskeletal traits

**DOI:** 10.1186/s12903-023-03504-y

**Published:** 2023-11-17

**Authors:** Farhad Sobouti, Sepideh Dadgar, Parsa Goleyjani, Vahid Rakhshan

**Affiliations:** 1 Dental Research Center, Mazandaran University of Medical Sciences, Sari, Iran; 2 Orthodontic Department, Faculty of Dentistry, Mazandaran University of Medical Sciences, Sari, Iran; 3 Private Practice, Sari, Iran; 4 Department of Dental Anatomy, Dental Faculty, Azad University of Medical Sciences, Tehran, Iran

**Keywords:** Cervical vertebral fusion, Atlas posterior arch deficiency, Ponticulus posticus (arcuate foramen, Sagittal foramen, Atlanto-occipital ligament calcification), Sella turcica bridging, Tooth size/shape/position/number anomalies, Concurrent abnormalities, Skeletal sagittal relationships, Vertical growth patterns, Incidental findings

## Abstract

**Introduction:**

Despite researchers' recent interest in identifying links between some dental and craniovertebral abnormalities, there are many important, unassessed gaps in our knowledge of this matter. In addition, previous samples were small. This large study aimed to examine, for the first time, the occurrence/severity of numerous dental and skeletal anomalies or variations and their correlations with each other and with growth patterns.

**Methods:**

This epidemiological study was conducted on pretreatment radiographs of 1194 patients from 3 cities (815 females). Skeletal sagittal skeletal relationships and vertical growth patterns were determined. The occurrence/severity were assessed for: cervical vertebral fusion (CVF), atlas posterior arch deficiency (APAD), ponticulus posticus (PP), sella turcica bridging (STB), hypodontia, oligodontia, hyperdontia, missing of maxillary laterals, microdontia, macrodontia, root dilaceration, odontoma, taurodontism, dental fusion, dental gemination, enamel pearl, permanent molar ankylosis, primary molar ankylosis, dens in dente, dens invaginatus, dental impaction, ectopic eruption, and dental transposition. Incidental findings were recorded as well. Concurrent anomalies, sex dimorphism, and correlations across variables were examined statistically, adjusting for the false discovery rate (α = 0.05).

**Results:**

Prevalence was calculated for 43 dentoskeletal traits/anomalies (22 abnormalities/variations [plus their severities/types] as well as 21 incidentally found traits/anomalies). Dental impaction may be more common in hyperdivergent and severer cases of sella bridging; also, primary molar ankylosis was associated with missing teeth. Dental impaction was associated only with STB and not with PP, APAD, or vertebral fusion. The only association observed among the four skeletal anomalies was seen between APAD and CVF. Merely the variables ‘sagittal skeletal relationships, vertical growth patterns, PP, and APAD’ showed sexual dimorphism; of these, only vertical growth pattern and APAD remained sexually dimorphic after adjusting for the FDR; still, the other two remained marginally significant and worth further evaluations. Sex dimorphism did not exist in concurrent abnormalities. The skeletal Class III was associated with the concurrent occurrence of craniovertebral, dental, and dentoskeletal abnormalities. Skeletal Class I was associated with fewer occurrences of concurrent dental anomalies. Vertical growth patterns were not associated with concurrent dental or dentoskeletal anomalies. However, the hyperdivergent pattern was associated with fewer cases of concurrent craniovertebral abnormalities.

**Conclusions:**

STB and hyperdivergent pattern were associated with dental impaction. However, APAD, CVF, or PP were not associated with dental impaction. APAD was associated with CVF. Sexual dimorphism existed conclusively in the case of vertical growth patterns and APAD. Concurrent abnormalities (dental, skeletal, and dentoskeletal) were associated with skeletal Class III.

## Introduction

Routine orthodontic imaging allows early diagnosis of numerous important skeletal and dental anomalies including sella turcica bridging, ponticulus posticus, atlas posterior arch deficiency, vertebral fusion, and countless dental abnormalities. Early identification of these features could help the identification of undetected syndromes or other serious conditions.

Sella turcica is a structure positioned on the intracranial surface of the sphenoid bone trunk. A frequent variation of sella turcica visible on lateral cephalograms is sella turcica bridging (STB), caused by over-calcification of the ligament between the sphenoid bone’s posterior and anterior clinoid processes or the sphenoid bone’s irregular growth; this anomaly has important clinical implications especially in neurosurgery [1–12]. Therefore, orthodontists can identify high-risk patients through their radiographic examinations.

Atlas arcuate foramen (also named atlas sagittal foramen, atlanto-occipital ligament calcification, or ponticulus posticus [PP]) is a frequent irregular bony protrusion, again visible on lateral cephalographs, and originating from the atlas vertebra and surrounding the upper articular artery entirely or partially, reaching the posterior arch of the atlas, which can lead to numerous clinical issues and again has neurosurgical implications [1–3, 6, 10, 12]. The posterior arch of the atlas itself can be deficient in about 5% to 14% of cases, called atlas posterior arch deficiency (APAD) which is yet another clinically meaningful skeletal anomaly of vertebrae visible in lateral cephalographs [2, 10, 13]. Vertebral deformities may be associated with clinical disorders such as migraines, shoulder pain, neck pain, vertigo, headache, or unconsciousness [14–17]. This is again relevant to orthodontics, since routine orthodontic radiographs can help finding high-risk individuals.

Many researchers consider some genetic mutations as well as the embryogenic origin shared between many dentoskeletal structures (i.e., the neural crest) responsible for numerous midface, dental, and skeletal anomalies [10, 11, 13, 18–21]. Therefore, it is expected to observe associations among many of them: Associations have been reported among many skeletal anomalies with dental anomalies such as skeletal malocclusion, maxillary canine impaction, hypoplastic enamel, hypodontia, submerged deciduous molars, and peg-shaped laterals [2, 7, 8, 10, 11]. Cervical vertebral anomalies may be associated with deformities in craniofacial structures, condyles, jaws, occlusion, and dental abnormalities [6, 9, 10, 14–17]. STB may be associated with several craniofacial or systemic developmental disorders and syndromes [11, 19, 22, 23] (but perhaps not necessarily with cleft palate [1]) as well as numerous local dental abnormalities like maxillary canine impaction, transposition, and hypodontia [10, 11, 13, 24, 25].

The abovementioned skeletal anomalies as well as many dentoalveolar anomalies several clinical implications. Many dental abnormalities can affect esthetics and/or function and thus need early diagnosis and proper treatment. This is why the abovementioned skeletal anomalies and their associations with some dental anomalies, especially sella turcica and canine impaction, are currently quite trendy and considered hot topics –as shown by a large number of studies that are published very recently [1–8, 12].

Nevertheless, numerous significant shortcomings have been identified in the literature. Firstly, most previous studies had relatively small samples, with sample sizes smaller than 100 subjects [1–8, 12]. Moreover, many aspects of dentoskeletal anomalies or traits seem to be understudied. These under-researched areas include the associations between skeletal abnormalities, the associations among many skeletal and/or dental abnormalities, and many dental anomalies that are not assessed before. Moreover, the numbers of articles published indicate that cervical vertebral anomalies (e.g., cervical vertebral fusion [CVF], PP, and APAD) have not been researched extensively like sella turcica bridging [1–3, 6, 10, 12, 13]. Hence, this large epidemiological study aimed to examine simultaneously, for the first time, vertical growth patterns and skeletal malocclusions, the occurrence and the magnitude of 4 craniovertebral abnormalities (STB, PP, APAD, and CVF), 18 dental anomalies, and the associations among all these variables. We also examined sex dimorphism as well as the factors associated with concurrent craniovertebral abnormalities, concurrent dental abnormalities, and all concurrent anomalies. Finally, we recorded the incidentally found items during the assessment of this large sample. We made sure not to introduce false negative errors (caused by our numerous hypotheses), using a proper method for false discovery rate (FDR) adjustment.

## Materials and methods

This retrospective analytical epidemiological study was performed on pre-treatment lateral cephalographs and panoramic radiographs of 1194 patients. The study population was selected from patients whose cephalometric and panoramic radiographic images were archived in selected radiology centers in three cities of ‘Babol, Amol, and Sari’, Iran between 2021 and 2022. The radiographs were archival and had been taken retrospectively for treatment purposes only. Therefore, no harm was imposed on any individuals (by the X-ray ionizing radiation) in this study, and the protocol was ethical. Since this study was performed on retrospectively taken anonymized human data, the need for informed consent to participate was waived by the Institutional Review Board of Mazandaran University of Medical Sciences, Sari, Iran (ethics code: IR.MAZUMS.REC.1400.248). The study was approved by the Mazandaran University of Medical Sciences, Sari, Iran. All methods were performed in accordance with the relevant guidelines and regulations (including the Declaration of Helsinki).

### Sample size

The sample size was pre-determined using the following formula and assuming conservative parameters within this formula: $n=({\mathrm{Z}}^{2}* p*(1- p) )/({\mathrm{d}}^{2})$ where Z = 1.96. In this formula, the *p* (prevalence) was assumed to be 0.5 as the most conservative prevalence, yielding the greatest sample within this formula. The parameter d (precision) was assumed to be 0.04, as a conservative precision yielding larger samples. The calculated sample size equaled 601 patients. It was doubled up to 1200 patients in order to ensure high test powers despite the multitude of hypotheses. Therefore, high powers for statistical analyses were expected despite many variables being considered at the same time.

### Eligibility criteria

The inclusion criteria comprised patients whose dental records included high-quality pre-treatment panoramic radiographs and lateral cephalograms, within which, all the teeth and surrounding structures were visible. All images must have been taken with the same radiology device. In terms of growth and maturity, the cervical vertebrae had to be in the CVM stage 5, in which there was a concavity in the lower margin of the cervical vertebrae C2, C3, C4 and at least one of the trunks of the third or fourth vertebrae was a vertical square. This stage indicates that the adolescent growth puberty spurt has likely ended [26, 27]. The minimum age of 14 years was chosen because, after puberty, the shape and size of sella turcica will not change significantly; and also because the canines’ development usually finishes at the ages of 12 or 13 years [10]. This minimum age might be as well proper for reliably diagnosing some other dental anomalies that depend on full development of the permanent teeth [28]. Patients with the following conditions were excluded from the study: Low-quality radiographs, a history of facial trauma, permanent tooth extraction, previous orthodontic treatment, cleft lip or palate or any other known craniofacial syndrome, any known systemic disease, endocrine imbalances, metabolic disorders, complex dental crown caries or restorations (which could interfere with the diagnosis of some coronal anomalies), and root canal treatment (which interferes with the diagnosis of some abnormalities such as taurodontism).

### Data collection

Patients’ sex was collected from their records. Their ages were not collected since it was well established that at least in young patients above 14 years old, aging might not have any significant role in the skeletal anomalies in question and in dental anomalies [6, 10, 29].

All radiographs in all centers had been taken using Vatech PaX-i Insight lateral cephalometric device (Vatech, Fort Lee, NJ, USA) with fixed magnification of 100% by an experienced technician. A radiologist first examined all cephalometric and panoramic radiographs. The radiologist approved all radiographs in terms of head position and contrast. An orthodontist then re-examined the cephalographs and panoramic radiographs for quality.

All the radiographic assessments and tracings were performed by a trained last-year dental student under the supervision of an orthodontist. A 15-inch monitor screen with a resolution of 1920 × 1080 was used to examine skeletal or dental anomalies. Lateral cephalographs were assessed to evaluate skeletal patterns and cranial and vertebral abnormalities. The Cephx software (ORCA Dental AI, Wilmington, Delaware, USA) was used for cephalometric tracings; this program’s validity was supported by Pamir and Naoumova [30] in 2020. All the skeletal anomalies and most of the dental abnormalities were re-examined later by two other observers: an experienced orthodontist jointly with an experienced radiologist. Also, the data were thoroughly checked for any inconsistencies or missing values by an epidemiologist and validated through various assessments. The following items were examined:

### Dental anomalies

Four types of dental anomalies were evaluated. For some of these, the type of the affected tooth or the unilateral/bilateral sidedness of the abnormality or the severity of the abnormality was assessed as well:

1. Abnormalities of the number of teeth: Hypodontia (congenital missing of six teeth or fewer, except for the third molars), oligodontia (congenital missing of more than six teeth except for the third molars), hyperdontia (accessory teeth except the third molars), and missing of maxillary laterals (considered separately from hypodontia).
2. Abnormalities of tooth sizes: microdontia (teeth considerably smaller than normal teeth, without different anatomic forms), and macrodontia (teeth markedly larger than normal teeth but with similar shapes).
3. Abnormalities of the shape of teeth: root dilaceration, odontoma, taurodontism, dental fusion, dental gemination, enamel pearl, permanent molar ankylosis, primary molar ankylosis, dens in dente, and dens invaginatus.
4. Abnormalities of the position of teeth: impaction (except the third molars), ectopic eruption, dental transposition.

### Vertical growth patterns

Steiner-based cephalometric radiography (SN-GoGn) was used and mandibular angle values were measured to describe growth patterns [31]: Hypodivergent: the SN-GoGn angle < 26.9°; Normal: SN-GoGn ranged between 26.9° and 37.1°; Hyperdivergent: SN-GoGn > 37.1°.

### Sagittal skeletal relationships

The skeletal classification was defined using the values of the angle between the jaws in the sagittal plane (SNA—SNB = ANB), according to the Steiner cephalometric standards [31]: Class III: ANB < 0°; Class I: 0° ≤ ANB ≤ 4°; Class II: ANB > 4°.

### Cervical vertebral fusion

**Fig. 1:**
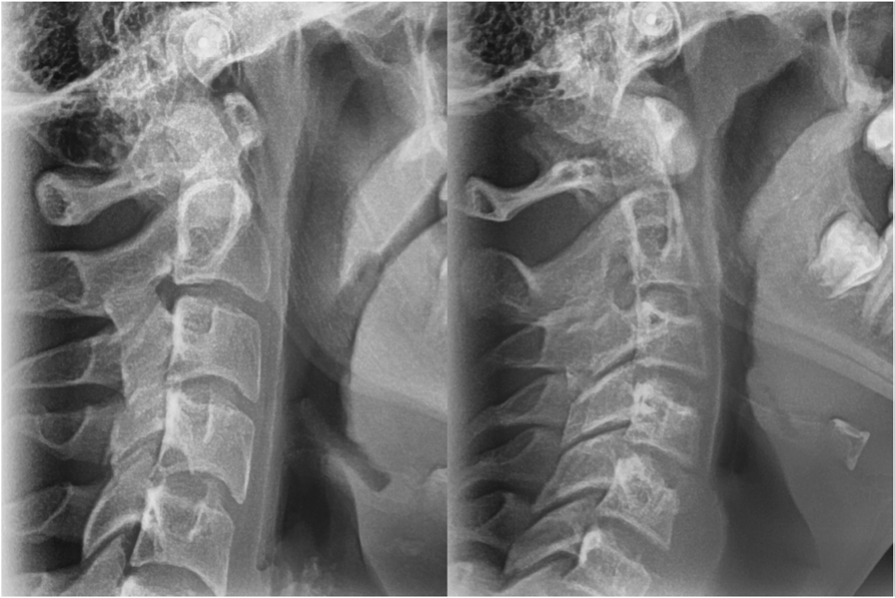
Two examples of cervical vertebral fusion

The morphology of the cervical spine including C1 (atlas), C2, and C3-C6 and their related anomalies were examined on the lateral cephalograms. In the normal cervical spine, the intervertebral spaces are seen as a radiolucent space of more than 1 mm. Surfaces that lacked such radiolucency indicated fusion, which could be continuous (complete) or discontinuous. In discontinuous fusion, articular surfaces were seen as separate radiopaque bone structures (Fig. 1) [14, 16, 32, 33].

### Ponticulus posticus

**Fig. 2:**
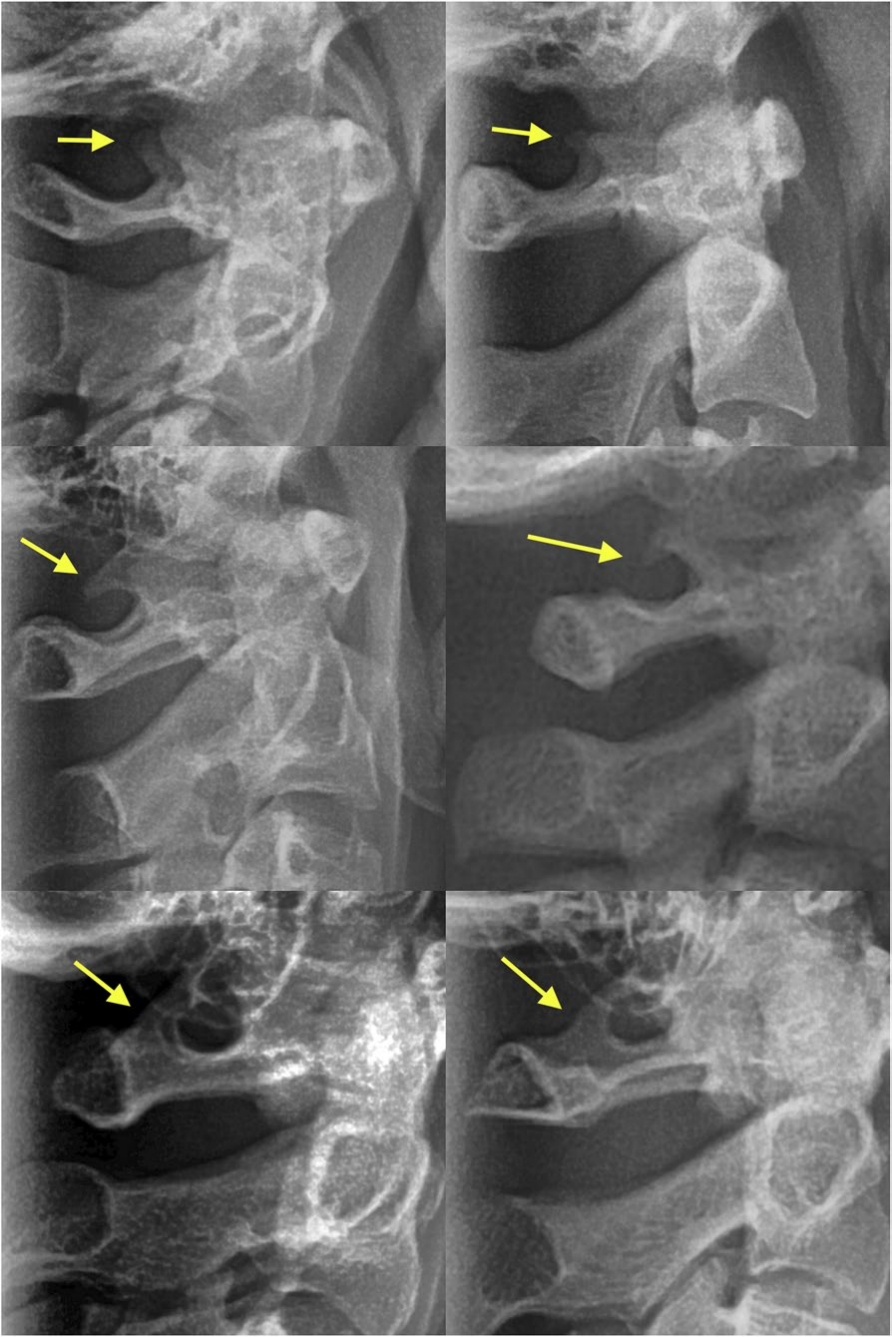
Examples of normal atlas without any ponticulus posticus (the top row), incomplete ponticulus posticus (the middle row), and complete ponticulus posticus (the bottom row). The arrows point to the bony emergences

The development extent of the atlas arcuate foramen also coined atlas ponticulus posticus (PP) was determined as the following types: No PP (the absence of any bony emergence), incomplete PP (a partial bony emergence), and complete PP (a complete bony bridge, Fig. 2) [6, 10].

### Atlas posterior arch deficiency (APAD)

**Fig. 3:**
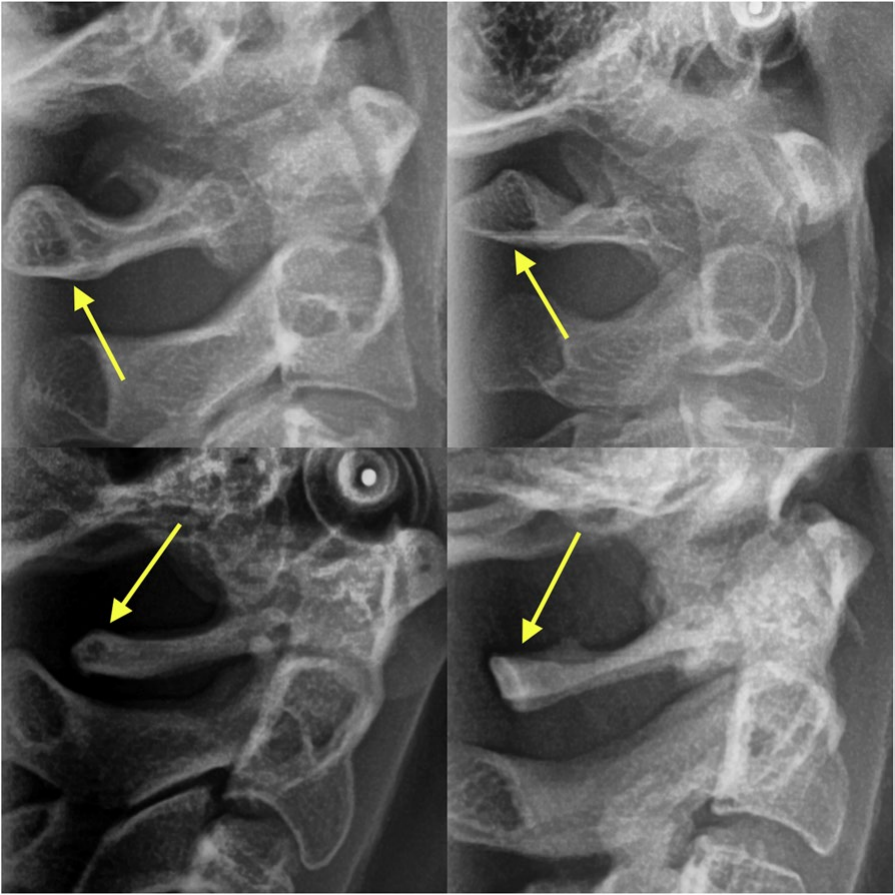
Examples of the normal (the top row) and deficient atlas posterior arches (the bottom row). The arrows indicate the posterior arches

Atlas posterior arch deficiency (APAD) was considered when the posterior atlas arch length was less than the mean of 4 mm. In fact, in posterior arch deficiency, we encounter a deficiency of the inner cortical layer of the posterior arch (Fig. 3) [2, 10, 13, 14, 16, 17, 32].

### Sella turcica bridging (calcification)

**Fig. 4:**
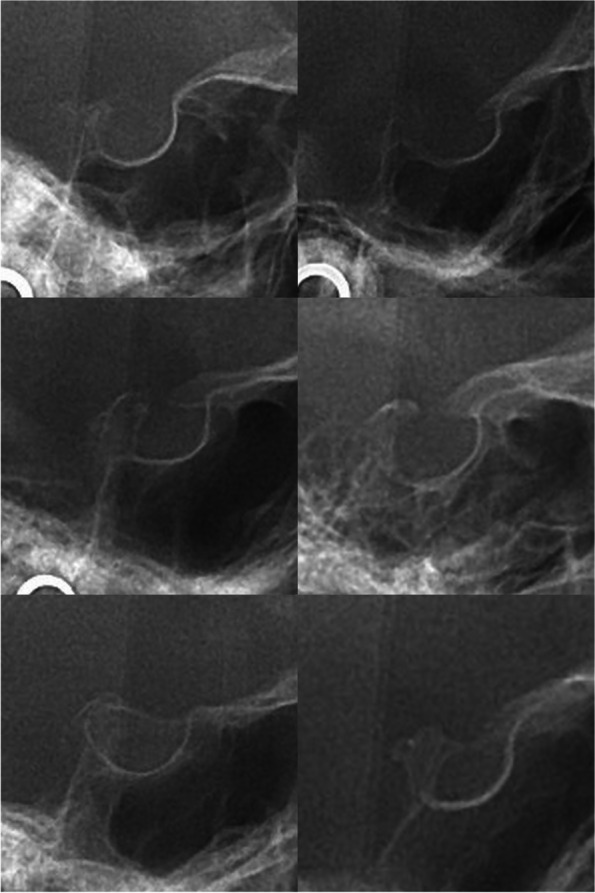
Examples of the normal sella turcica (the top row), type II / partial bridging of the sella turcica (the middle row), and type III / complete calcification of the sella turcica (the bottom row)

Sella turcica is a u-shaped structure extending from the dorsum sella (ds) to the tuberculum sella (ts) [6, 10, 11, 13]. Based on these, the following measurements can be estimated: (1) the distance between ts to ds head as the interclinoid distance or sella length: (2) the longest distance from the farthest point on the inner surface of the posterior sella contour to the ts as the sella diameter or maximum posterior sella length [6, 10, 11, 13]. According to the Leonardi standard classification, the following types can be considered for the bridging of the sella turcica: Type I or normal sella turcica with a sella length equal to or longer than 3/4 of the sella diameter. Type II or partial calcification having a length 3/4 of sella diameter. Type III or complete calcification, in which the anterior and posterior clinoid distance is less than 1 mm or only the sella diaphragm is visible (Fig. 4) [6, 10, 11, 13].

### Incidental findings

All lateral cephalographs and panoramic radiographs of all patients were carefully re-examined jointly by an orthodontist and a radiologist for any incidental findings outside of the assessed variables not expected when the radiograph was indicated. The assessment of all the radiographs for other findings showed 23 different incidental findings. Two of these were added to the study variables for further examination. All the lateral cephalographs and panoramic radiographs of the 1194 patients were re-examined carefully by both the orthodontist and radiologist for the presence of all 21 incidental findings in all radiographs.

### Concurrent abnormalities

The numbers of concurrent skeletal anomalies, dental anomalies, and all anomalies (regardless of their skeletal or dental types) were calculated.

### Intrarater and interrater reliabilities

#### Initial assessment

After about two months, the same observer re-assessed 19 randomly selected cases in terms of all the variables except a few ones, which were re-assessed by the orthodontist instead (i.e., cephalometric tracings and CVF). According to the Cronbach Alpha, there were perfect interrater agreements in terms of ANB (Alpha = 1.0, *P* = 0.000) and S–N^Go-Gn (Alpha = 1.0, *P* = 0.000). The presence and severity of STB were identical in both assessments (all the 19 cases showed no bridging in both assessments). The presence and severity of PP as well showed a perfect agreement (Kappa = 1.0, *P* = 0.000). The presence of APAD as well had a perfect agreement (Kappa = 1.0, *P* = 0.000). Both assessments similarly showed no instances of hyperdontia, hypodontia, microdontia, macrodontia, odontoma, taurodontism, dental fusion, gemination, enamel pearl, dental impaction, and transposition. There were perfect agreements in terms of oligodontia (Kappa = 1.0, *P* = 0.000) and ectopic eruption (Kappa = 1.0, *P* = 0.000).

#### Secondary assessment

After a year (in 2023), 100 new cases were randomly selected and re-analyzed by the orthodontist in terms of all the variables. According to the Cronbach Alpha, the intrarater agreement was perfect in terms of ANB (Alpha = 1.0, *P* = 0.000) and S–N^Go-Gn (Alpha = 1.0, *P* = 0.000). The inter-rater agreements for the presence and severity of STB were excellent (Kappa = 0.912, *P* = 0.000). The presence and severity of PP had an excellent agreement (Kappa = 0.945, *P* = 0.000). The presence of APAD had an excellent agreement (Kappa = 0.928, *P* = 0.000). The presence of vertical fusion had a perfect agreement (Kappa = 1.0, *P* = 0.000). The repeated assessments showed perfect agreements in terms of the presence of hypodontia, oligodontia, macrodontia, odontoma, dental impaction, transposition, permanent molar ankylosis, and dens evaginatus (all Kappa values = 1.0, *P* = 0.000). For some traits, none of the assessments resulted in any instances; these were hyperdontia, microdontia, taurodontism, dental fusion, gemination, enamel pearl, ectopic eruption, primary molar ankylosis, the missing of maxillary laterals, and dens in dente.

### Statistical analysis

Intraobserver and interobserver agreements were measured twice: (1) once after about two months, on all variables within 19 cases; and (2) for a second time, after about one year, again on all variables within 100 other cases. 

Descriptive statistics and 95% confidence intervals were calculated for the prevalence of different variations and anomalies. Sex dimorphism was assessed using a chi-square test or a Fisher exact test. The correlations among the occurrences of skeletal and dental anomalies as well as their types or severities were examined using a Spearman correlation coefficient and a chi-square test of SPSS 25 (IBM, Armonk, NY, USA). The effect sizes were calculated as the Spearman Rho values. Associations between concurrent abnormalities with skeletal growth patterns were evaluated using a chi-square test. The *q* values were computed by adjusting the calculated *P* values for the false discovery rate (FDR) using the Benjamini–Hochberg method. The level of significance was set at 0.05.

## Results

A total of 1194 patients were included in the study. Of them, 815 and 372 were females and males, respectively; the sex of 7 cases was not recorded. There was no missing data regarding any anomalies, traits, or variations. The prevalence of different skeletal or dental features and anomalies and their Wilson 95% confidence intervals are presented in Table 1.

**Table 1:** Net frequencies and prevalence percentages of various parameters (including skeletal Classes, vertical growth patterns, and dentoskeletal anomalies or traits) as well as 95% CIs for the prevalence percentages. The sample size was 1194 patients

**Parameter**	**Levels**	**Frequency (%)**	**Wilson 95% CI (%)**	
**Sagittal Skeletal Relationship**	**Class I**	782 (65.49)	62.75	68.14
	**Class II**	368 (30.82)	28.27	33.50
	**Class III**	44 (3.69)	2.76	4.91
**Vertical Growth Pattern**	**Hypodivergent**	81 (6.78)	5.49	8.35
	**Normal**	686 (57.45)	54.63	60.23
	**Hyperdivergent**	427 (35.76)	33.09	38.52
**Sella Turcica Bridging**	**Class I (No bridging)**	978 (81.91)	79.62	83.99
	**Class II (Partial bridging)**	159 (13.32)	11.51	15.36
	**Class III (Complete bridging)**	57 (4.77)	3.70	6.14
**Ponticulus Posticus**	**Class I (No calcification)**	973 (81.49)	79.19	83.59
	**Class II (Partial calcification)**	167 (13.99)	12.13	16.07
	**Class III (Complete calcification)**	54 (4.52)	3.48	5.85
**Atlas Posterior Arch Deficiency**		136 (11.39)	9.71	13.32
**Cervical Vertebral Fusion**	**None**	942 (78.89)	76.49	81.11
	**Incomplete**	151 (12.65)	10.88	14.65
	**Complete**	101 (8.46)	7.01	10.17
**Hyperdontia**		9 (0.75)	0.40	1.43
**Hypodontia**		29 (2.43)	1.70	3.47
**Oligodontia**		23 (1.93)	1.29	2.87
**Microdontia**		2 (0.17)	0.05	0.61
**Macrodontia**		12 (1.01)	0.58	1.75
**Odontoma**		9 (0.75)	0.40	1.43
**Taurodontism (Prevalence)**		8 (0.67)	0.34	1.32
**Taurodontism (Severity)**	**Hypo**	5 (0.42)	0.18	0.98
	**Meso**	2 (0.17)	0.05	0.61
	**Hyper**	1 (0.08)	0.01	0.47
**Dental Fusion**		4 (0.34)	0.13	0.86
**Gemination**		2 (0.17)	0.05	0.61
**Enamel pearl**		3 (0.25)	0.09	0.74
**Impaction**		30 (2.51)	1.77	3.56
**Transposition**		44 (3.69)	2.76	4.91
**Ectopic eruption**		6 (0.50)	0.23	1.09
**Primary molar ankylosis**		9 (0.75)	0.40	1.43
**Maxillary lateral missing (Prevalence)**		8 (0.67)	0.34	1.32
**Maxillary lateral missing (Laterality)**	**Unilateral**	5 (0.42)	0.18	0.98
	**Bilateral**	3 (0.25)	0.09	0.74
**Permanent molar ankylosis (Prevalence)**		3 (0.25)	0.09	0.74
**Permanent molar ankylosis (Tooth Type)**	**Maxillary 6**	2 (0.17)	0.05	0.61
	**Mandibular 6**	1 (0.08)	0.01	0.47
**Dens in Dente**		2 (0.17)	0.05	0.61
**Dens evaginatus**		2 (0.17)	0.05	0.61

### Sex dimorphism

Sex dimorphism existed only in the case of sagittal skeletal relationships (females tended to have more cases of Class II), vertical growth patterns (men had more hypodivergent growth patterns, while females had more normal and hyperdivergent growths), PP (women had more normal cases, while men more partial PP cases), and APAD (females had a greater proportion of normal cases, Table 2). After adjusting for the FDR, only two of these four variables remained statistically significant (vertical growth pattern and APAD) while the other two became marginally significant (Table 3).

**Table 2:** Sex dimorphism in various skeletal Classes, vertical growth patterns, or traits and anomalies, calculated using the chi-square or Fisher tests. The *q* values are calculated by adjusting the *P* values for the FDR using the Benjamini–Hochberg method. The sample size was 1194 patients

**Trait**	**Level**	**Sex**		***P***	***FDR-adjusted q***
		**Female (%)**	**Male (%)**		
**Sagittal Skeletal Relationship**	**Class I**	513 (66.02)	264 (33.98)	**0.013**	0.088
	**Class II**	273 (74.59)	93 (25.41)		
	**Class III**	29 (65.91)	15 (34.09)		
**Vertical Growth Pattern**	**Hypodivergent**	41 (51.25)	39 (48.75)	**0.002**	**0.027**
	**Normal**	478 (69.99)	205 (30.01)		
	**Hyperdivergent**	296 (69.81)	128 (30.19)		
**Sella Turcica Bridging**	**Class I (normal)**	660 (67.97)	311 (32.03)	0.454	0.751
	**Class II**	116 (72.96)	43 (27.04)		
	**Class III**	39 (68.42)	18 (31.58)		
**Ponticulus Posticus**	**Class I (normal)**	680 (70.25)	288 (29.75)	**0.009**	0.081
	**Class II**	97 (58.43)	69 (41.57)		
	**Class III**	38 (71.70)	15 (28.30)		
**Atlas Posterior Arch Deficiency**	**Absent**	739 (70.11)	315 (29.89)	**0.002**	**0.027**
	**Present**	76 (57.14)	57 (42.86)		
**Vertebral Fusion**	**Absent**	643 (68.7)	293 (31.3)	0.112	0.497
	**Incomplete**	111 (73.51)	40 (26.49)		
	**Complete**	61 (61.00)	39 (39.00)		
**Hyperdontia**	**Absent**	810 (68.76)	368 (31.24)	0.473^**f**^	0.751
	**Present**	5 (55.56)	4 (44.44)		
**Hypodontia**	**Absent**	799 (68.94)	360 (31.06)	0.184	0.497
	**Present**	16 (57.14)	12 (42.86)		
**Oligodontia**	**Absent**	796 (68.38)	368 (31.62)	0.177^**f**^	0.497
	**Present**	19 (82.61)	4 (17.39)		
**Microdontia**	**Absent**	813 (68.61)	372 (31.39)	1.0^**f**^	1.0
	**Present**	2 (100)	0		
**Macrodontia**	**Absent**	809 (68.85)	366 (31.15)	0.209^**f**^	0.513
	**Present**	6 (50.00)	6 (50.00)		
**Odontoma**	**Absent**	810 (68.76)	368 (31.24)	0.473^**f**^	0.751
	**Present**	5 (55.56)	4 (44.44)		
**Taurodontism (Prevalence)**	**Absent**	809 (68.62)	370 (31.38)	1.0^**f**^	1.0
	**Present**	6 (75.00)	2 (25.00)		
**Taurodontism (Severity)**	**Absent**	809 (68.62)	370 (31.38)	0.782	1.0
	**Hypo**	4 (80.00)	1 (20.00)		
	**Meso**	1 (50.00)	1 (50.00)		
	**Hyper**	1 (100)	0		
**Dental Fusion**	**Absent**	812 (68.64)	371 (31.36)	1.0^**f**^	1.0
	**Present**	3 (75.00)	1 (25.00)		
**Gemination**	**Absent**	813 (68.61)	372 (31.39)	1.0^**f**^	1.0
	**Present**	2 (100)	0		
**Enamel pearl**	**Absent**	812 (68.58)	372 (31.42)	0.556^**f**^	0.751
	**Present**	3 (100)	0		
**Impaction**	**Absent**	796 (68.8)	361 (31.2)	0.524	0.751
	**Present**	19 (63.33)	11 (36.67)		
**Transposition**	**Absent**	789 (69.03)	354 (30.97)	0.163	0.497
	**Present**	26 (59.09)	18 (40.91)		
**Ectopic eruption**	**Absent**	811 (68.67)	370 (31.33)	1.0^**f**^	1.0
	**Present**	4 (66.67)	2 (33.33)		
**Primary molar ankylosis**	**Absent**	807 (68.51)	371 (31.49)	0.287^**f**^	0.554
	**Present**	8 (88.89)	1 (11.11)		
**Maxillary lateral missing (Prevalence)**	**Absent**	809 (68.62)	370 (31.38)	1.0^**f**^	1.0
	**Present**	6 (75.00)	2 (25.00)		
**Maxillary lateral missing (Laterality)**	**Absent**	809 (68.62)	370 (31.38)	0.134	0.497
	**Unilateral**	5 (100)	0		
	**Bilateral**	1 (33.33)	2 (66.67)		
**Permanent molar ankylosis (Prevalence)**	**Absent**	814 (68.75)	370 (31.25)	0.233^**f**^	0.524
	**Present**	1 (33.33)	2 (66.67)		
**Permanent molar ankylosis (Tooth Type)**	**Absent**	814 (68.75)	370 (31.25)	0.284^**f**^	0.554
	**Maxillary 6**	1 (50.00)	1 (50.00)		
	**Mandibular 6**	0	1 (100)		
**Dens in Dente**	**Absent**	815 (68.78)	370 (31.22)	0.098^**f**^	0.497
	**Present**	0	2 (100)		
**Dens evaginatus**	**Absent**	814 (68.69)	371 (31.31)	0.529^**f**^	0.751
	**Present**	1 (50.00)	1 (50.00)		

*FDR* False discovery rate

^f^The superscript f denotes the use of Fisher exact test instead of the chi-square test. The rest of *P* values are calculated using the chi-square test

**Table 3:** The results of the Spearman correlation coefficient. N for each correlation was 1194

**Variable**		**Skeletal Classes**	**Vertical Growth Patterns**	**STB**	**PP**	**APAD**	**CVF**	**Hyperdontia**	**Hypodontia**	**Oligodontia**	**Microdontia**	**Macrodontia**	**Odontoma**
**Vertical Growth pattern**	**Rho**	0.089											
	***P***	**0.002**											
**STB**	**Rho**	-0.010	-0.020										
	***P***	0.731	0.485										
**PP**	**Rho**	0.026	0.048	0.022									
	***P***	0.364	0.100	0.440									
**APAD**	**Rho**	-0.113	-0.086	0.046	-0.022								
	***P***	**0.000**	**0.003**	0.113	0.449								
**Cerebral Vertebral Fusion**	**Rho**	-0.020	-0.337	0.006	0.020	0.213							
	***P***	0.483	**0.000**	0.849	0.498	**0.000**							
**Hyperdontia**	**Rho**	0.087	0.021	0.035	0.030	-0.031	-0.023						
	***P***	**0.003**	0.458	0.223	0.304	0.281	0.423						
**Hypodontia**	**Rho**	0.040	0.066	0.023	0.013	0.046	-0.039	-0.014					
	***P***	0.164	**0.022**	0.437	0.659	0.111	0.177	0.635					
**Oligodontia**	**Rho**	-0.015	0.043	0.030	-0.052	-0.012	-0.045	-0.012					
	***P***	0.600	0.135	0.297	0.075	0.682	0.121	0.673					
**Microdontia**	**Rho**	-0.029	0.014	-0.019	0.031	0.050	0.035	-0.004	-0.006	-0.006			
	***P***	0.309	0.621	0.508	0.289	0.086	0.225	0.902	0.823	0.843			
**Macrodontia**	**Rho**	-0.039	0.040	0.060	0.042	0.043	0.009	-0.009	-0.016	-0.014	-0.004		
	***P***	0.181	0.167	**0.037**	0.143	0.136	0.764	0.762	0.583	0.626	0.887		
**Odontoma**	**Rho**	-0.024	-0.003	0.007	-0.018	-0.031	0.030	-0.008	-0.014	-0.012	-0.004	-0.009	
	***P***	0.411	0.913	0.811	0.542	0.281	0.302	0.793	0.635	0.673	0.902	0.762	
**Taurodontism**	**Rho**	0.010	0.016	-0.038	-0.014	-0.029	-0.042	-0.007	0.054	0.063	-0.003	-0.008	-0.007
	***P***	0.726	0.590	0.184	0.633	0.309	0.145	0.805	0.063	**0.029**	0.908	0.775	0.805
**Dental Fusion**	**Rho**	-0.013	-0.033	-0.027	-0.028	-0.021	0.010	-0.005	-0.009	-0.008	-0.002	-0.006	-0.005
	***P***	0.663	0.250	0.349	0.342	0.473	0.731	0.862	0.752	0.779	0.935	0.840	0.862
**Gemination**	**Rho**	-0.029	-0.024	-0.019	-0.019	-0.015	-0.021	-0.004	-0.006	-0.006	-0.002	-0.004	-0.004
	***P***	0.309	0.417	0.508	0.502	0.612	0.468	0.902	0.823	0.843	0.954	0.887	0.902
**Enamel pearl**	**Rho**	0.010	0.033	-0.023	0.025	-0.018	-0.026	-0.004	-0.008	-0.007	-0.002	-0.005	-0.004
	***P***	0.742	0.255	0.417	0.386	0.534	0.373	0.880	0.785	0.808	0.943	0.861	0.880
**Impaction**	**Rho**	-0.015	0.072	0.109	0.049	0.010	-0.029	0.048	-0.025	0.016	-0.007	-0.016	0.233
	***P***	0.606	**0.013**	**0.000**	0.088	0.735	0.312	0.098	0.382	0.570	0.820	0.577	**0.000**
**Transposition**	**Rho**	0.026	-0.040	-0.046	-0.019	0.070	0.007	0.034	0.027	0.005	-0.008	-0.020	0.137
	***P***	0.363	0.172	0.115	0.510	**0.016**	0.811	0.236	0.353	0.865	0.782	0.496	**0.000**
**Ectopic eruption**	**Rho**	0.053	-0.056	0.031	0.030	0.012	0.016	-0.006	-0.011	-0.010	-0.003	-0.007	-0.006
	***P***	0.070	0.054	0.29	0.302	0.684	0.574	0.831	0.699	0.731	0.920	0.805	0.831
**Primary molar ankylosis**	**Rho**	0.075	0.075	0.031	-0.013	-0.001	-0.018	-0.008	0.552	0.058	-0.004	-0.009	-0.008
	***P***	**0.010**	**0.009**	0.288	0.652	0.979	0.529	0.793	**0.000**	**0.044**	0.902	0.762	0.793
**Maxillary lateral missing**	**Rho**	0.066	0.035	0.037	-0.009	0.003	-0.014	-0.007	0.521	-0.012	-0.003	-0.008	-0.007
	***P***	**0.022**	0.232	0.196	0.757	0.921	0.628	0.805	**0.000**	0.691	0.908	0.775	0.805
**Permanent molar ankylosis**	**Rho**	-0.003	-0.029	-0.023	0.025	0.035	0.011	-0.004	0.318	-0.007	-0.002	-0.005	-0.004
	***P***	0.931	0.320	0.417	0.386	0.231	0.692	0.880	**0.000**	0.808	0.943	0.861	0.880
**Dens in Dente**	**Rho**	-0.029	-0.050	0.041	-0.019	0.050	-0.021	-0.004	-0.006	-0.006	-0.002	-0.004	-0.004
	***P***	0.309	0.087	0.158	0.502	0.086	0.468	0.902	0.823	0.843	0.954	0.887	0.902
**Dens evaginatus**	**Rho**	-0.029	-0.024	0.031	0.091	-0.015	-0.021	-0.004	-0.006	-0.006	-0.002	-0.004	-0.004
	***P***	0.309	0.417	0.280	**0.002**	0.612	0.468	0.902	0.823	0.843	0.954	0.887	0.902
**Variable**		**Taurodontism**	**Fusion**	**Gemination**	**Enamel pearl**	**Impaction**	**Transposition**	**Ectopic eruption**	**Primary molar ankylosis**		**Maxillary lateral missing**	**Permanent molar ankylosis**	**Dens in Dente**
**Dental Fusion**	**Rho**	-0.005											
	***P***	0.869											
**Gemination**	**Rho**	-0.003	-0.002										
	***P***	0.908	0.935										
**Enamel pearl**	**Rho**	-0.004	-0.003	-0.002									
	***P***	0.887	0.920	0.943									
**Impaction**	**Rho**	0.052	-0.009	-0.007	-0.008								
	***P***	0.070	0.748	0.820	0.781								
**Transposition**	**Rho**	0.038	-0.011	-0.008	0.079	0.224							
	***P***	0.185	0.695	0.782	**0.006**	**0.000**							
**Ectopic eruption**	**Rho**	-0.006	-0.004	-0.003	0.233	-0.011	0.112						
	***P***	0.840	0.887	0.920	**0.000**	0.694	**0.000**						
**Primary molar ankylosis**	**Rho**	-0.007	-0.005	-0.004	-0.004	-0.014	0.034	-0.006					
	***P***	0.805	0.862	0.902	0.880	0.629	0.236	0.831					
**Maxillary lateral missing**	**Rho**	-0.007	-0.005	-0.003	-0.004	-0.013	0.038	-0.006	0.586				
	***P***	0.816	0.869	0.908	0.887	0.649	0.185	0.840	**0.000**				
**Permanent molar ankylosis**	**Rho**	-0.004	-0.003	-0.002	-0.003	-0.008	-0.010	-0.004	-0.004		-0.004		
	***P***	0.887	0.920	0.943	0.931	0.781	0.735	0.902	0.880		0.887		
**Dens in Dente**	**Rho**	-0.003	-0.002	-0.002	-0.002	-0.007	-0.008	-0.003	-0.004		-0.003	-0.002	
	***P***	0.908	0.935	0.954	0.943	0.820	0.782	0.920	0.902		0.908	0.943	
**Dens evaginatus**	**Rho**	-0.003	-0.002	-0.002	-0.002	-0.007	-0.008	-0.003	-0.004		-0.003	-0.002	-0.002
	***P***	0.908	0.935	0.954	0.943	0.820	0.782	0.920	0.902		0.908	0.943	0.954

Rho, Spearman correlation coefficient

Significant *P* values in bold

*STB* Sella turcica bridging, *PP* Ponticulus posticus, *APAD* Atlas posterior arch deficiency, *CVF* Cervical vertebral fusion

### Associations among the variables

The Spearman correlation coefficient showed some significant correlations among some anomalies or traits (Table 3). After correcting for the FDR, 17 significant correlations were identified (Table 4).

**Table 4:** The *q* values calculated by adjusting the *P* values of the Spearman correlation coefficient (presented in Table 3) for the false discovery rate, using the Benjamini–Hochberg method

***FDR-adjusted q values***	Malocclusion	Vertical Growth	STB	PP	APAD	CVF	Hyperdontia	Hypodontia	Oligodontia	Microdontia	Macrodontia	Odontoma
Vertical Growth pattern	**0.037**											
STB	0.957	0.957										
PP	0.957	0.708	0.957									
APAD	**0.000**	**0.049**	0.756	0.957								
CVF	0.957	**0.000**	0.957	0.957	**0.000**							
Hyperdontia	**0.049**	0.957	0.957	0.957	0.957	0.957						
Hypodontia	0.912	0.253	0.957	0.957	0.756	0.912	0.957					
Oligodontia	0.957	0.834	0.957	0.627	0.957	0.777	0.957					
Microdontia	0.957	0.957	0.957	0.957	0.656	0.957	0.957	0.957	0.957			
Macrodontia	0.912	0.912	0.393	0.851	0.834	0.957	0.957	0.957	0.957	0.957		
Odontoma	0.957	0.957	0.957	0.957	0.957	0.957	0.957	0.957	0.957	0.957	0.957	
Taurodontism	0.957	0.957	0.912	0.957	0.957	0.851	0.957	0.580	0.320	0.957	0.957	0.957
Dental Fusion	0.957	0.957	0.957	0.957	0.957	0.957	0.957	0.957	0.957	0.957	0.957	0.957
Gemination	0.957	0.957	0.957	0.957	0.957	0.957	0.957	0.957	0.957	0.957	0.957	0.957
Enamel pearl	0.957	0.957	0.957	0.957	0.957	0.957	0.957	0.957	0.957	0.957	0.957	0.957
Impaction	0.957	0.171	**0.000**	0.656	0.957	0.957	0.708	0.957	0.957	0.957	0.957	**0.000**
Transposition	0.957	0.912	0.756	0.957	0.201	0.957	0.957	0.957	0.957	0.957	0.957	**0.000**
Ectopic eruption	0.604	0.514	0.957	0.957	0.957	0.957	0.957	0.957	0.957	0.957	0.957	0.957
Primary molar ankylosis	0.138	0.131	0.957	0.957	0.979	0.957	0.957	**0.000**	0.450	0.957	0.957	0.957
Maxillary lateral missing	0.253	0.957	0.949	0.957	0.957	0.957	0.957	**0.000**	0.957	0.957	0.957	0.957
Permanent molar ankylosis	0.957	0.957	0.957	0.957	0.957	0.957	0.957	**0.000**	0.957	0.957	0.957	0.957
Dens in Dente	0.957	0.656	0.909	0.957	0.656	0.957	0.957	0.957	0.957	0.957	0.957	0.957
Dens evaginatus	0.957	0.957	0.957	**0.037**	0.957	0.957	0.957	0.957	0.957	0.957	0.957	0.957

***FDR-adjusted q values***	Taurodontism	Fusion	Gemination	Enamel pearl	Impaction	Transposition	Ectopic eruption	Primary molar ankylosis	Lateral missing	Permanent molar ankylosis	Dens in Dente	
Dental Fusion	0.957											
Gemination	0.957	0.957										
Enamel pearl	0.957	0.957	0.957									
Impaction	0.604	0.957	0.957	0.957								
Transposition	0.912	0.957	0.957	0.092	**0.000**							
Ectopic eruption	0.957	0.957	0.957	**0.000**	0.957	**0.000**						
Primary molar ankylosis	0.957	0.957	0.957	0.957	0.957	0.957	0.957					
Maxillary lateral missing	0.957	0.957	0.957	0.957	0.957	0.912	0.957	**0.000**				
Permanent molar ankylosis	0.957	0.957	0.957	0.957	0.957	0.957	0.957	0.957	0.957			
Dens in Dente	0.957	0.957	0.957	0.957	0.957	0.957	0.957	0.957	0.957	0.957		
Dens evaginatus	0.957	0.957	0.957	0.957	0.957	0.957	0.957	0.957	0.957	0.957	0.957	

Significant *q* values in bold

*STB* Sella turcica bridging, *PP* Ponticulus posticus, *APAD* Atlas posterior arch deficiency, *CVF* Cervical vertebral fusion

The sagittal skeletal relationship was weakly but significantly associated with vertical growth pattern, APAD, hyperdontia, primary molar ankylosis, and missing maxillary laterals. Regarding the association between skeletal relationship and vertical growth patterns, there were respectively 48, 27, and 6 cases with Classes I, II, and III in the hypodivergent group; there were respectively 487, 177, and 22 cases with Classes I, II, and III in the normal group; and there were respectively 427, 164, and 16 cases with Classes I, II, and III in the hyperdivergent group (chi-square, *P* = 0.000). Regarding the association between APAD and malocclusion, in the Class I, II, and III skeletal relationships, there were 113, 11, and 12 cases with APAD (showing a tendency for APAD to occur more frequently in Classes II or III compared to Class I, chi-square *P* = 0.000).

The vertical growth pattern was weakly correlated with APAD, CVF, hypodontia, dental impaction, and primary molar ankylosis. Regarding the association between APAD and vertical growth pattern, there were 28, 64, and 44 cases in the groups Hypodivergent, Normal, and Hyperdivergent, respectively (showing that APAD was more common in hypodivergent followed by hyperdivergent, chi-square *P* = 0.000). Regarding the association between vertical growth and dental impaction, there were 2, 10, and 18 cases in the groups Hypodivergent, Normal, and Hyperdivergent, respectively (showing that dental impaction was more common in hyperdivergent cases, chi-square *P* = 0.017).

Sella turcica bridging was correlated weakly with dental impaction and macrodontia: there were 17, 8, and 5 cases in Classes I (normal), II, and III of sella turcica bridging, respectively (showing that dental impaction was more common in Class III STB > Class II > Class I, chi-square *P* = 0.000).

Dental impaction was not correlated with ponticulus posticus, APAD, or vertebral fusion; these results were also confirmed using the chi-square test (*P* > 0.1).

Cervical vertebral fusion was correlated with APAD: there were 81, 0, and 55 cases with APAD in the CVF stages ‘healthy, incomplete, and complete’, respectively (showing that APAD was much more frequent in completely formed APAD cases, chi-square *P* = 0.000). Except this, there was no other significant association among the skeletal anomalies APAD, STB, PP, and CVF (chi-square and Spearman *P* values ≥ 0.1).

Overall, there was no strong correlation and only 3 were moderate (absolute Spearman coefficients about 0.5). These were only the correlations between primary molar ankylosis with either of the anomalies ‘hypodontia and maxillary lateral missing’ or between hypodontia and maxillary lateral missing (Table 3).

### Incidental findings

Prevalence and 95% confidence intervals are reported for the incidental findings in different head and neck areas (Table 5).

**Table 5:** The net frequency and prevalence percentage of incidental findings assessed in all the 1194 patients as well as Wilson 95% CIs for the percentages

**Dentoalveolar region in both jaws**	**Frequency (%)**	**Wilson 95% CI (%)**	
Retained primary tooth fragments	9 (0.75)	0.40	1.43
Root dilaceration	23 (1.93)	1.29	2.87
Rarefying osteitis	27 (2.26)	1.56	3.27
Cemento-osseous dysplasia	4 (0.34)	0.13	0.86
Odontogenic cyst	3 (0.25)	0.09	0.74
Enostosis (focal sclerosis)	6 (0.50)	0.23	1.09
External root resorption	19 (1.59)	1.02	2.47
Internal root resorption	2 (0.17)	0.05	0.61
**Airway region**			
Adenoid hypertrophy	69 (5.78)	4.59	7.25
Nasal polyp	3 (0.25)	0.09	0.74
Retention pseudocyst	41 (3.43)	2.54	4.63
Turbinate hypertrophy	11 (0.92)	0.52	1.64
Sinus pneumatization	30 (2.51)	1.77	3.56
Antrolith	2 (0.17)	0.05	0.61
**Hard/soft tissues in panoramic radiographs**			
Osteoma	5 (0.42)	0.18	0.98
Calcified stylohyoid ligament	3 (0.25)	0.09	0.74
Mandibular body fracture	1 (0.08)	0.01	0.47
Dystrophic calcification of lymph nodes	6 (0.50)	0.23	1.09
Degenerative findings in condyle	15 (1.26)	0.76	2.06
Repaired condylar fracture	2 (0.17)	0.05	0.61
Condylar hypoplasia	3 (0.25)	0.09	0.74

### Concurrent abnormalities

#### *Craniovertebral abnormalities*

Of the 1194 cases, 434 (36.3%), 155 (13.0%), 19 (1.6%), and 6 (0.5%) had respectively 1, 2, 3, or 4 skeletal abnormalities. Thus, concurrent skeletal anomalies (at least 2 abnormalities together in the same individual) existed in 15.07% of the sample. These numbers were 415, 284, 100, 10, 6 in women and 162, 148, 53, 9, 0 in men. Concurrent skeletal anomalies were not associated with sex (Table 6). However, they tended to occur more in skeletal Class III malocclusion cases and less in hyperdivergent cases (*P* and FDR-adjusted *q* < 0.05, Table 6).

**Table 6:** The net frequency (and prevalence, %) of cases with only 1 abnormality versus cases with concurrent (2 or more) abnormalities. Cases with zero anomalies are not presented or compared. The *P* value is calculated using the chi-square test. The *q* values are calculated by adjusting the *P* values for the FDR using the Benjamini–Hochberg method

**Anomaly types**	**Associated Factors**	**Number of Anomalies**		***P***	***FDR-adjusted q***
		**1**	** ≥ 2**		
**Craniovertebral**	**Female**	284 (71.00)	116 (29.00)	0.892	0.892
	**Male**	148 (70.48)	62 (29.52)		
**Dental**	**Female**	83 (81.37)	19 (18.63)	0.528	0.595
	**Male**	44 (77.19)	13 (22.81)		
**Dentoskeletal**	**Female**	288 (64.00)	162 (36.00)	0.277	0.415
	**Male**	138 (59.74)	93 (40.26)		
**Craniovertebral**	**Class I**	281 (68.37)	130 (31.63)	**0.007**	**0.021**
	**Class II**	139 (78.53)	38 (21.47)		
	**Class III**	14 (53.85)	12 (46.15)		
**Dental**	**Class I**	91 (86.67)	14 (13.33)	**0.001**	**0.005**
	**Class II**	34 (72.34)	13 (27.66)		
	**Class III**	3 (37.50)	5 (62.50)		
**Dentoskeletal**	**Class I**	280 (61.40)	176 (38.60)	**0.001**	**0.005**
	**Class II**	139 (69.15)	62 (30.85)		
	**Class III**	9 (32.14)	19 (67.86)		
**Craniovertebral**	**Hypodivergent**	45 (67.16)	22 (32.84)	**0.012**	**0.028**
	**Normal**	248 (67.21)	121 (32.79)		
	**Hyperdivergent**	141 (79.21)	37 (20.79)		
**Dental**	**Hypodivergent**	7 (63.64)	4 (36.36)	0.188	0.339
	**Normal**	67 (84.81)	12 (15.19)		
	**Hyperdivergent**	54 (77.14)	16 (22.86)		
**Dentoskeletal**	**Hypodivergent**	42 (60.00)	28 (40.00)	0.499	0.595
	**Normal**	248 (61.23)	157 (38.77)		
	**Hyperdivergent**	138 (65.71)	72 (34.29)		

Significant *P* or *q* values in bold

*FDR* False discovery rate

#### *Dental abnormalities*

Of the patients, 128 (10.7%), 21 (1.8%), 9 (0.8%), 2 (0.2%), had 1, 2, 3, or 4 dental anomalies, respectively (713, 83, 10, 7, 2 women and 315, 44, 11, 2, 0 men); therefore, concurrent dental anomalies (at least 2 abnormalities) were observed in 2.68% of the sample. Sex or vertical growth patterns did not affect the prevalence of concurrent dental abnormalities (Table 6). However, concurrent dental anomalies occurred less in skeletal Class I cases and more in Class III cases (*P* and FDR-adjusted *q* < 0.05, Table 6).

#### *Dentoskeletal concurrent anomalies*

When counting the number of dentoskeletal abnormalities in each case (regardless of the types of anomalies [craniovertebral or dental]), it was observed that 509 (42.6%), 428 (35.8%), 193 (16.2%), 43 (3.6%), 19 (1.6%), 1 (0.1%), and 1 (0.1%) cases had 1, 2, 3, 4, 5, and 6 abnormalities (365, 288, 122, 25, 13, 1, and 1 females and 141, 138, 70, 17, 6, 0, and 0 males), amounting to 57.37% concurrent dentoskeletal abnormalities. Sex dimorphism was not observed in concurrent dentoskeletal anomalies, which were also not associated with vertical growth pattern (Table 6). Nonetheless, dentoskeletal anomalies were observed more in skeletal Class III malocclusion cases (*P* and *q* < 0.05, Table 6).

## Discussion

Some of the associations, although hypothetically possible, are of relatively low prevalence. Hence, the chance of identifying significant correlations is meagre when associating events of lower prevalence. Also, this brings to attention the need to better phenotype populations when exploring associations, as there is a good chance that mixing up or including any case may dilute any potential meaningful correlations. Dental craniofacial research should focus on phenotyping subgroups of patients that may benefit from specific diagnostic or management approaches.

Our finding regarding the positive correlation between the severity of STB and dental impaction was similar to many previous studies on canine impaction [10, 11, 13, 34–36] and in contrast to few others on canine impaction [37]. STB has been found to be associated with some other dental anomalies (like number anomalies, lateral or premolar aplasia, root dilaceration, altered directions of dental eruption, or dental displacement) [7, 8, 10, 22–25, 35, 36, 38–43], vertebral anomalies, craniofacial anomalies, and even cancers [5, 10, 22–24, 33, 38–40]. Such associations may be explained by the role of neural crest cells as the originator of numerous structures and the developmental role of HOX or Homeobox genes [13]. However, we could not find an association between STB and the four craniovertebral anomalies. The prevalence of STB in our study was about 18% which was much smaller than many other studies, even than their control groups, reporting percentages such as 50% [37] but similar to or greater than some others [38, 40]. Consistent with many studies, STB did not show sex dimorphism in our study [6, 11, 29]; still, some studies have shown a positive role for sex [5]. Unlike some earlier research [29], we could not identify any links between STB and sagittal skeletal relationship. Since both STB and skeletal relationship share some similar genetic mechanisms involved in bone formation and development [10, 11, 13, 18–21], it might be reasonable to expect the existence of some links between the two. Nevertheless, such overlapped genetic modifiers may not be the sole etiology for either of these. Therefore, it is also possible to see a variety of patterns of associations between these two features, depending on numerous other known and unknown confounding factors.

When it comes to studies on the associations between PP with skeletal or dental anomalies, the literature is much scarcer than that of STB. We could not find any significant associations between PP with numerous skeletal or dental anomalies (except dens evaginatus), which was in contrast to studies showing associations between PP and dental impaction [6, 10, 13]. Our PP prevalence falls within the range reported earlier [6, 10, 24, 44]. Also the sex dimorphism observed in this study (i.e., PP was more common in men) was similar to a previous study [44], but in contrast to some other ones [6, 45]. The lack of associations between PP and STB was also seen in certain other studies (similar to the present study) [6, 13], but not in others [45]. Such partial associations observed in some studies and the lack of them reported by some others may be related to a range of factors including, but not limited to, the partially shared genetic etiologies [10, 11, 13, 18–21] as well as other factors such as the age ranges of the populations studied, the sex distributions of the subjects, and other known and unknown factors. Of course, each of such deductions needs its own research. Moreover, statistical factors may matter as well; for instance, the sample sizes of many of the studies were not large, and this might have led to nonsignificant results (false negatives).

Studies on APAD are even scarcer. Unlike another study which found a link between APAD and canine impaction [10], our study and Ghadimi et al*.* [10] could not identify meaningful connections (at least definitively). Still, APAD was associated with vertical fusion, which can be due to their similar origins. No studies in this regard existed to compare our results. APAD was more common in men, unlike another study showing no sex dimorphism [46]. This as well needs more research. As stated above, each of these dental and skeletal anomalies may share some similar genetic etiologies [10, 11, 13, 18–21]. These can justify the co-occurrences of some of such abnormalities.

In the present study, cervical vertebral fusion was linked only to vertical growth patterns and APAD, but not other dental or skeletal variations or anomalies. Similar to another study [46], we did not observe sex dimorphism or an association between CVF and skeletal relationship. Still, another study found a correlation between vertebral fusion and jaw relationships [32]. It should be noted however that lateral cephalographs are not an optimum tool to examine vertical fusion, as they can yield a considerable false positive error [47].

Canine impaction was not influenced by sex. This was in contrast to a literature review concluding that it is more common in women [11, 48], but similar to some other studies [11]. Similarly, hypodontia might be more prevalent in women [49]. However, this study could not find such results. These need more examinations. The controversies observed in each of the findings can root in numerous factors such as methodological differences in data collection, sample sizes, ethnic backgrounds, and many other known and unknown factors.

Potential associations between dental abnormalities and traits with the skeletal classes are rare. In this regard, Fernandez et al. [50] reported an association between the Class III malocclusion and microdontia. However, Ashoori et al. [51] did not find such an association. Both studies did not report an association between hyperdontia and skeletal malocclusion [50, 51]. Ashoori et al. [51] observed associations between the skeletal malocclusions with some dental anomalies or traits; according to them, skeletal Class II cases were associated with shoveling of the anterior teeth, talon cusps on the canines, canine distal accessory ridges, and accessory cusps on the first premolars; they reported some other associations as well [51].

Some factors might limit this study. This research with its large sample and its numerous variables was a rather difficult task to complete. In this regard, artificial intelligence algorithms might speed up the process of detecting anomalies. Currently there are proposed programs that may estimate cervical maturation [52] or identify lateral cephalometric landmarks [53]. Similar programs can be developed to identify anomalies in the craniovertebral or dental areas. Nevertheless, their unsupervised use in research is not possible unless they are proven as accurate as or even better than experienced clinical experts, i.e., the gold standard. Using CBCT instead of lateral cephalography and panoramic radiography could improve the diagnosis accuracy [4]. Nevertheless, including 1200 extra-large-field CBCTs covering the whole skull and jaws was impossible and also ethically unacceptable if those CBCTs were not adequately justified. This is because practically no therapeutic or diagnostic approaches necessitate such vast fields of view. And therefore, due to the ALARA guidelines, most archival CBCTs have much smaller fields of view. So, the only way someone could do such research would be to take such CBCTs for research purposes prospectively; and this is not ethically approvable. The same reason, i.e., the X-ray hazard, forced us (and most other researchers) to sample only from a group of patients with retrospectively available therapeutic radiographs; obviously, it was not possible to sample randomly and prospectively from the general population, due to the ethical issues associated with X-ray and its dangers. However, even if it was practically possible to obtain 1200 archival CBCTs with very large fields of view, there would be yet another problem: available 3D imaging –especially with large fields of view that can encompass the whole skull and vertebral structures– is likely from a subsample of patients with a higher likelihood for complex craniofacial problems (so that the CBCT imaging was properly indicated). This would imply non-representativeness. Additionally, 2D imaging technology used represents what most orthodontists in the world are daily exposed to. Finally, although it is not ideal like 3D imaging, it is still valid. It should be noted that the discrepancies between studies is least likely caused by the 2D versus 3D methods of imaging, since most studies in this regard are on 2D radiographs. Therefore, differences in the results may be attributed to other methodological and sampling differences. The fact that our sample was consisted of dental patients could limit the generalizability of our findings to dental patients only. However, this is a limitation shared by all retrospective radiographic studies. Another factor limiting the generalizability of this (and any other) research was the ethnic background of the assessed population; however, at least, sampling from three different cities in this study would allow more diverse sub-ethnic backgrounds to be included. It might be argued that a control group is required to find out whether the frequency of anomalies found is unusual. However, epidemiological studies are performed using cross-sectional designs and not case–control or retrospective cohort designs. This is because a cross-sectional study can provide a snapshot of the population, while this is not the case with case–control and retrospective cohort studies that are goal-oriented. Moreover, when the sample size is large enough, various characteristics are already included within the final sample, allowing for statistical comparisons and tests. For instance, patients with a skeletal Class I relationship only with crowding could somehow serve as a control sample. Another argument is that orthodontic patients might bias the results compared to randomly selected individuals across the country. In our country like many other ones, there is no center that has retrospectively taken orthodontic radiographs from completely random individuals (such as school children) only for the sake of research; not to mention that this might not be even ethical. Therefore, we were limited to orthodontic patients. Still, the current sample represents an array of patients typically seen in an orthodontic office. An advantage of this study was that we did not limit the maximum age as an inclusion criterion. Although it might not affect the occurrence of anomalies, it still could influence skeletal patterns [54]. Therefore, enrolling merely children might skew the results related to skeletal patterns. A broad age range is beneficial because it reduces data skewness and improves the generalizability of the findings to a broader age range beyond only children or young adults. Some may argue that a large number of variables and so many hypotheses may be considered an undirected fishing expedition. However, the assessment of associations is a part of epidemiological studies. Additionally, if the familywise error is corrected, there is no major concern for false positive errors caused by an excessive number of hypotheses. Another critique might be the small correlation coefficients obtained in this study, which are not clinically useful. However, the lack of strong correlations is itself a result. Besides, orthodontics is not merely about clinical findings; it is also concerned with scientific findings such as prevalence rates or associations between different traits. A small effect size is itself a proper scientific finding when the sample size is large. It shows a decisive lack of correlation, which is something worthwhile.

## Conclusions

The prevalence and 95% CIs of 22 dental anomalies/variations and their types as well as 21 incidental findings were calculated. A summary of major findings could be that: dental impaction may be more common in hyperdivergent and severer cases of sella bridging; also, primary molar ankylosis was associated with missing teeth. Dental impaction was associated only with STB and not with PP, APAD, or vertebral fusion. The only association observed among the four skeletal anomalies was seen between APAD and CVF. Merely the variables ‘sagittal skeletal relationships, vertical growth patterns, PP, and APAD’ showed sexual dimorphism; of these, only vertical growth pattern and APAD remained sexually dimorphic after adjusting for the FDR; still, the other two remained marginally significant and worth further evaluations.

Sex dimorphism did not exist in concurrent abnormalities. The skeletal Class III was associated with the occurrence of concurrent craniovertebral, dental, and dentoskeletal abnormalities. Skeletal Class I was associated with fewer occurrences of concurrent dental anomalies. Vertical growth patterns were not associated with concurrent dental or dentoskeletal anomalies. However, the hyperdivergent pattern was associated with fewer cases of concurrent craniovertebral abnormalities.

## Data Availability

The data are available from the corresponding author upon request.
